# Prioritising and incentivising productivity within indicator-based approaches to Research Impact Assessment: a commentary

**DOI:** 10.1186/s12961-023-01082-7

**Published:** 2023-12-18

**Authors:** Simon Deeming, Alexis Hure, John Attia, Michael Nilsson, Andrew Searles

**Affiliations:** 1 Hunter Medical Research Institute, Kookaburra Circuit, New Lambton Heights, Newcastle, NSW Australia; 2 School of Medicine and Public Health, University of Newcastle, Callaghan, Newcastle, NSW Australia; 3 Department of Medicine, John Hunter Hospital, Hunter New England Local Health District, New Lambton Heights, Newcastle, NSW Australia; 4 Centre for Rehab Innovations, College of Health, Medicine and Wellbeing, University of Newcastle, Callaghan, Newcastle, NSW Australia

**Keywords:** Productivity, Medical research, Health research, Australia, Research assessment, Research impact

## Abstract

Research Impact Assessment (RIA) represents one of a suite of policies intended to improve the impact generated from investment in health and medical research (HMR). Positivist indicator-based approaches to RIA are widely implemented but increasingly criticised as theoretically problematic, unfair, and burdensome. This commentary proposes there are useful outcomes that emerge from the process of applying an indicator-based RIA framework, separate from those encapsulated in the metrics themselves. The aim for this commentary is to demonstrate how the act of conducting an indicator-based approach to RIA can serve to optimise the productive gains from the investment in HMR. Prior research found that the issues regarding RIA are less about the choice of indicators/metrics, and more about the discussions prompted and activities incentivised by the process. This insight provides an opportunity to utilise indicator-based methods to purposely optimise the research impact. An indicator-based RIA framework specifically designed to optimise research impacts should: focus on researchers and the research process, rather than institution-level measures; utilise a project level unit of analysis that provides control to researchers and supports collaboration and accountability; provide for prospective implementation of RIA and the prospective orientation of research; establish a line of sight to the ultimate anticipated beneficiaries and impacts; Include process metrics/indicators to acknowledge interim steps on the pathway to final impacts; integrate ‘next’ users and prioritise the utilisation of research outputs as a critical measure; Integrate and align the incentives for researchers/research projects arising from RIA, with those existing within the prevailing research system; integrate with existing peer-review processes; and, adopt a system-wide approach where incremental improvements in the probability of translation from individual research projects, yields higher impact across the whole funding portfolio.

Optimisation of the impacts from HMR investment represents the primary purpose of Research Impact policy. The process of conducting an indicator-based approach to RIA, which engages the researcher during the inception and planning phase, can directly contribute to this goal through improvements in the probability that an individual project will generate interim impacts. The research project funding process represents a promising forum to integrate this approach within the existing research system.

## Introduction

### Policy context

The health and medical research (HMR) conducted within Australia over the past five decades, has made an exceptional contribution to the improved health, well-being and longevity of Australian and international populations during this period [1, 2]. A long list of exemplars includes the success of tobacco control policy in reducing the prevalence and acceptability of smoking [3], reduction in the prevalence of neural tube defects, particularly for Indigenous Australians, resulting from increased periconceptional folate consumption [4], development of an artificial heart valve [5], advancement of the understanding of the role of antibodies in the immune system [6], the development of in vitro fertilisation [7], and more recently, the potential elimination of cervical cancer through improved screening, development, and successful uptake, of the Human Papilloma Virus (HPV) vaccine [8].

Despite challenging national fiscal budgets amid slower economic growth internationally, health and medical research (HMR) has continued to attract considerable public investment and philanthropic support. Internationally, the United States (US$50.5 billion per annum in 2015), Europe (US$26.9 billion) and Japan (US$17.0 billion) direct significant funds into HMR [9–12].

Optimisation of the impact from this funding remains high on the policy agenda for many OECD nations, including Australia [13–17]. However, several inter-related economic imperatives accentuate these expectations. First, fiscal policy in response to recent economic crises has seen public debt surge, placing greater pressure on post-crisis fiscal budgets [18, 19]. As a consequence, there is a greater requirement to both maximise the benefits realised from public expenditure on HMR, when compared to alternative public investments, and minimise any wastage [20–25]. Secondly, the demand for public health services continues to rise due to the increased prevalence of chronic diseases, the ageing demographic in western nations, and the higher cost of technological solutions [26, 27]. Finally, Australia’s position as a small open economy within the international economic system is founded on specialisation to the nation’s comparative and competitive advantages [28]. Consequently, for Australia’s economy to remain productive, it is imperative that opportunities to commercialise the knowledge generated from HMR are optimised through innovations in medical services, pharmaceuticals, and medical devices [27]. These pressing imperatives affirm that the primary objective of research impact policy is to increase the impacts generated from investment in HMR.

In response to these imperatives, numerous policies have been introduced globally to improve research translation and impact. New pools of funding were established and dedicated to translational goals, such as Australia’s Medical Research Future Fund (MRFF), the United States’ Clinical and Translational Science Awards (US CTSA) [13] and the United Kingdom’s National Institute for Health Research (UK NIHR) targeted funding schemes [29]. By example, Australia’s new MRFF aims to ‘transform health and medical research and innovation to improve lives, build the economy and contribute to health system sustainability’ [30]. New institutions were established focussed on the translation of HMR e.g. the Australian Health Research Translation Centres [31], the United States’ Patient Centred Outcomes Research Institute and Clinical and Translational Science Institutes [32], the United Kingdom’s NIHR Biomedical Research Centres and Collaborations for Leadership in Applied Health Research and Care (CLAHRCs) in the United Kingdom [33] and the Canadian Institutes of Health Research [11]. Complimentary policy initiatives include those relating to research careers [34, 35], research quality and waste [21, 36, 37], commercialisation [38, 39], implementation [40–42], and patient/consumer engagement [43–45]. In this light, the assessment of the impact from research funding can be understood as a complementary policy designed to improve the productive impacts of HMR [46].

Definitions in this field remain disputed, but for the purpose of this paper we adopt the broad International School definition of Research Impact Assessment (RIA) being the ‘growing field of practice that is interested in science and innovation, research ecosystems and the effective management and administration of research funding’ [47]. Within this context, Research Impact Assessment Frameworks (RIAFs) provide a conceptual framework and methods against which the translation and impact of health and medical research can be assessed [48–50].

Australia’s independent Medical Research Institutes (iMRIs) represent the research setting for this study. The iMRIs provide a valuable setting for RIA policy and practice discussions, due to their pure focus on research, rather than a divided focus between education (universities) and/or healthcare responsibilities e.g., clinician/public health researchers. Approximately 70 iMRIs operate within Australia, supporting the more than 10,000 researchers across the spectrum of diseases, populations and research stages [51, 52]; as such, a significant proportion of the nation’s HMR funding passes through this setting. In addition, the majority of mission statements and strategic plans for Australian iMRIs identify the realisation of community benefits, most commonly health and economic impacts, as their ultimate objective [46], which aligns closely with the objectives for research impact policies.

### Indicator-based approaches to RIA

The research methods commonly applied within RIAFs, comprise indicator/metrics-based methods; experimental, statistical, and data mining methods; systems analysis methods; textual and oral methods; economic methods; bibliometrics; and evidence synthesis methods [46, 50, 53–55]. Aside from case studies, indicator/metrics-based methods applied retrospectively at the institution or program level represent the most common approach to RIA [2, 56, 57].

Indicator/metrics-based methods utilise logic models, theory of change and similar frameworks to identify metrics or indicators, making explicit the assumed cause and effect relationships with research outputs, outcomes, and impacts [55, 58]. This study relates explicitly to the indicator-based RIAFs grounded in logic models. The mechanism underpinning this method involves construction of a program logic model, or equivalent [59], which identifies the research activities being conducted, the research outputs, users, and the impacts generated [58, 60]. The process of constructing such a model serves to demonstrate the anticipated causal pathway and provides a basis for the selection of appropriate indicators to demonstrate progression from research activities through to the realisation of impact [58, 60, 61]. Perceived advantages of this method includes its appeal to intuition and relative transparency [49, 58], and consistency with common government performance evaluation methods for public expenditure [62]. Appropriate indicators can be selected from standardised published lists, such as Washington University’s *Becker list* [63], the US CTSA’s *Common Metrics* [64], or RAND’s *100 Metrics to Assess and Communicate the Value of Biomedical Research* [65], or designed with reference to guides for high quality indicators [66]. The majority of indicator-based RIAFs are designed to provide information suitable for *Accountability*, *Advocacy* and to a lesser degree, insights for *Management/Learning and feedback*/*Allocation* [57].

### Criticisms of indicator-based approaches to RIA

Indicator-based approaches to RIA are attracting increasingly strident criticism. The criticisms include:

- Research complexity: Retrospective review of research, commonly practiced with indicator-based methods, wrongly infers a deterministic linear research process along the research spectrum to implementation, in contrast to the complex reality of research pathways [58, 67, 68].
- Attribution and causation, amid long time lags: The time lag to the generation of final impacts from conduct and inception of the HMR, can be as long as 17 + years [38, 69]. Verification of causal links and/or the proportion of attribution to a specified HMR investment is accordingly dependent on the effect of numerous other potentially confounding influences [47, 68, 70, 71].
- Data limitations: Quantitative analyses of RIA are constrained by the breadth, consistency, validity and availability of data. The surge and decline in bibliometrics exemplifies this issue. Electronic databases, such as Researchfish®, Vertigo Ventures^®^, Overton^®^, and new open-source formats, are improving the collation of non-academic impact data, but these datasets remain partial, expensive (excluding the open sources initiatives) and were not designed to guide the optimisation of research translation, nor to analyse how to improve the productive impacts from research [56].
- Subjectivity: Impacts from research are subjective, they may generate positive outcomes for some at the expense of others, or the same impact could be perceived as positive or negative by different stakeholders [55].
- Unpredictability: it has also been argued that HMR is by nature random, fraught with anomalies, and unpredictable [72, 73].
- Perverse incentives: generic indicators risk irrelevance across disciplines or worse, generate incentives that countermand productivity [74]. For example, bibliometric impact indicators encouraged ‘salami’ publishing [75] and academic publication indicators can incentivise revelation of intellectual property in advance of the optimal time for a commercialisation pathway [76]. In extreme cases, the established incentives can encourage misconduct by researchers [77] or institutions [78].
- Administrative burden: The potential administrative burden that arises from the conduct of RIA represents a real and significant challenge and consumes resources (time and money) that may otherwise be directed to HMR [14, 51, 79]. The displacement of researchers’ time due to RIA requirements, also often goes unrecognised and unvalued [76]. The conduct of RIA potentially reduces productivity, unless the holistic process serves to generate equivalent or greater gains.

The aim of this commentary is to demonstrate how the process of conducting an indicator-based approach to RIA, containing specific principles, can serve to optimise the productive gains from the investment in HMR, such as the realisation of commercial opportunities, improvements to the health service and a reduction in research waste, and thereby align the assessment process with the fundamental productivity-focussed objectives for RIA policy.

## Methods

The aim was addressed through a synthesis of the insights drawn from our prior studies. These included an overview of the policy drivers and policy initiatives [2], a scoping literature review to identify specified purposes for RIA [57], and a document review to evaluate the capacity of alternative Research Impact Assessment Frameworks (RIAFs) to realise these objectives [57]. To ground the study within real-world health and medical research, insights were also drawn from our prior qualitative research, which examined stakeholders’ perspectives of RIA policy and practice within a research-focussed setting, Australia’s iMRIs [76]. The results from these methods were subsequently synthesised to establish guiding principles for an indicator-based RIAF that explicitly prioritises the productivity objective of research translation and impact policy.

## Definitions

The following definitions have been adopted for this paper to provide clarity. Some have been adapted to reflect specific choices in line with the focus on productivity and indicator-based RIAFs. The definitions are:

- Optimisation—economic optimisation seeks to maximise the objective function, e.g., social welfare/utility, given the constraints on the pursuit of this objective [80]. In layperson terms, and for this thesis, the process of economic optimisation seeks to maximise the health, economic and social impacts from HMR given the constraints of funding limits, system-wide rigidities, ethical parameters, etc.
- Productivity—the relationship between inputs of resources and the output/outcomes realised from the specified resources [80].
- Research waste—funded research that produces outputs that are either unusable or under-utilised due to avoidable errors in study selection, study design, research conduct, publication, and/or reporting [21].
- Research activity—activities necessary to conduct health and medical research, where research is defined as ‘creative work undertaken on a systematic basis in order to increase the stock of knowledge, including knowledge of man, culture and society, and the use of this stock of knowledge to devise new applications’ [81].
- Research translation—the dynamic flow of the knowledge created by a research activity from generation to utilisation. This definition applies across the full research spectrum [82] and acknowledges that research translation can be multidirectional and non-sequential [60, 83].
- Research outputs—the knowledge deliverables produced by research activity e.g. peer-reviewed papers, presentations, contributions to collaborative endeavours, guidelines, education, etc.
- Research outcomes/Interim impacts—the demonstrable effect at a static point and time within the research system, when research outputs transform to research outcomes/interim impacts following utilisation by the ‘next’ user along the pathway to final impact.
- Implementation—the process of putting recommendations derived from research evidence into practice.
- Final impacts—following implementation of research-generated knowledge, the demonstrable positive effect upon human health, quality of life, society, the economy, culture, national security, or the environment.

### Guiding principles for an indicator-based productivity-focussed RIAF

The premise of this commentary is that the objective for all research translation and impact policy is to improve the productive impacts from the investment in HMR. This implies that the primary objective for RIA and RIAFs should also prioritise productivity. The following principles seek to demonstrate how the process of applying an indicator-based RIA can guide and optimise research activity and, so contribute to this goal. In summary, the principles are:

- A focus upon researchers and the research process
- A unit of analysis that provides control for researchers and supports both collaboration and accountability
- Prospective implementation of RIA enabling the prospective orientation of research
- A line of sight to the ultimate anticipated beneficiaries and benefits (pathway to impact)
- Inclusion of process metrics/indicators that provide for interim targets on the pathway to the final impacts
- A logic model that embeds ‘next’ users and generates outcomes from outputs along the pathway
- Alignment with a potential incentive mechanism within the existing research system to motivate researchers/research teams to optimise the impacts from their research and reduce waste
- Alignment with existing peer-review processes and norms
- An over-arching objective to enhance productivity and maximise the value from all funded HMR, through incremental improvements in the probability of translation for individual research projects.

The following explains the rationale underpinning each principle.

### A focus upon researchers and the research process

The capacity for impact assessment, and impact measurement more explicitly, to affect research behaviour is accepted [60, 66, 76, 84–87], but this influence is not inevitable. The activities of researchers reflect existing incentives, mainly to publish peer-reviewed papers and realise research grants, but also to teach or conduct their health practice [76]. Institutional assessment frameworks intended to encourage research impact can only be effective to the extent that these signals translate to meaningful incentives for researchers. For example, the Australian Research Council’s *Engagement and Impact Assessment* (ARC EIA) framework for Australian universities is aggregated by field of research and institution [88]. The accompanying requirement for relevance across disparate disciplines, such as the humanities and computer science, significantly constrains both the methods and the granularity of any assessment techniques. However, the more generic and institutional the indicators, the less influence these incentives have on research activity.

Institutional frameworks may encourage the introduction of supporting mechanisms, for example via research translation training, but rely upon transition through institutional systems e.g., promotion criteria, to generate meaningful incentives at the researcher-level. Existing impact frameworks/methods, such as Glover’s *Economic Impact Assessments* [89], the ARC’s *EIA* [90] and Australian Academy of Technology and Engineering’s proposed *Research Engagement for Australia* [91] represent assessments that could only directly influence research activity via additional mechanisms. Furthermore, the ability of impact assessment to incentivise research translation and impact are relative to the existing incentives. For example, if the generation of impacts are not proportionately valued in funding application weights, track records, team capabilities, and/or grant review panels, nor wider commitments, such as teaching loads, then the capacity for assessment to change behaviour will be limited. RIAFs that do not acknowledge the research/researcher perspective may collate information suitable for institutional accountability/audit but will fail to influence individual research behaviour and productivity.

**Fig. 1:**
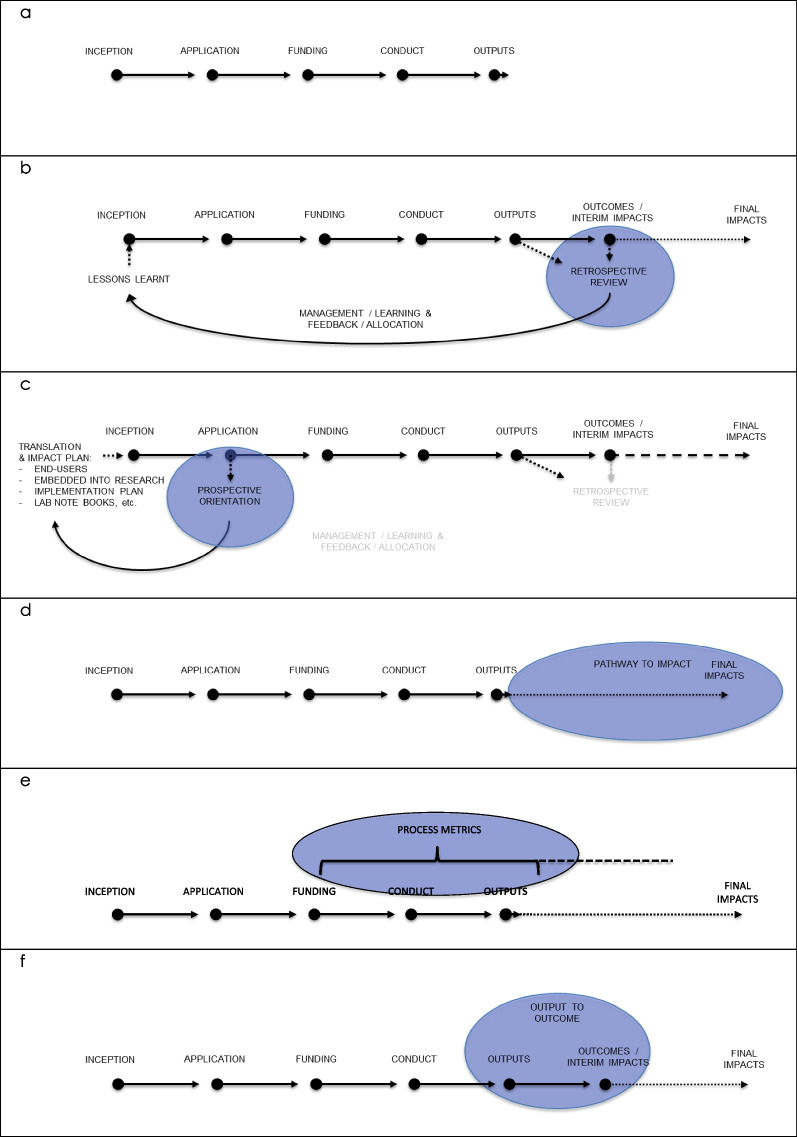
Generic subprocess of a research study demonstrating the guiding principles

Figure 1 demonstrates how this principle re-imagines RIA through a researcher’s lens. Figure 1a, adapted from Trochim et al. [92], reviewers of the US CTSA Program, represents a simple schematic of the research process from this perspective. It commences with inception of the research question and study design, progresses through application and, if successful, funding, to the conduct of the research and production of academic outputs or knowledge products. The subsequent principles are presented from this researcher perspective.

### Unit of analysis

The minimum unit of analysis should be determined by the incentives conveyed to researchers and the alignment of these incentives with improvements in productivity and the realisation of health/economic/social impacts. For example, individual key performance indicators may weaken incentives to collaborate, implying that the unit of analysis should be team-based [76]. The upper threshold should remain below the level at which governance cannot significantly inform research activity, that is, at a point where researchers retain sufficient control to be motivated and accountable for the outcomes. This implies that research projects represent the optimal unit of analysis. The definitional boundaries of a research project can be determined from the perspective of the lead Chief Investigator.

Project-level RIA provides both greater control and accountability for researchers to effect productive change, in contrast to RIA mechanisms operating at the program or institution level. For other specified objectives for RIA, such as *Accountability* and *Management*, *Learning and feedback*, project-level indicators can subsequently be aggregated for retrospective research program or institution-level assessment [93]. Project-level RIA also provides transparency to successes, barriers, constraints and failures for all funded and reported research and not just the positive examples commonly cited within case studies [76].

### Prospective implementation of RIA enabling the prospective orientation of research

Of the 25 RIAFs reviewed in our prior study, only four were designed to be implemented prospectively, with the majority assuming retrospective assessment of research impact [94]. For most frameworks the outputs and outcomes from research, as portrayed in Fig. 1b, are retrospectively collated following completion of the research. This allows management to analyse the data/information and for the available insights to inform subsequent decisions regarding resource allocation, recruitment, etc. This feedback loop represents an important method to improve the productivity from HMR. However, Fig. 1c demonstrates that the speed of translation and ensuing productivity can be improved further if critical decisions are made at inception of the research. The prospective implementation of RIA provides the capacity to identify key opportunities to improve translation before the research is conducted, thereby improving the probability that research evidence is translated.

Compared to a retrospective analysis, the prospective implementation of research also minimises data collection costs, thereby addressing another concern for researchers and institutions [60, 76].

### A line of sight to the ultimate anticipated beneficiaries & benefits (pathway to impact)

Only a few of the existing RIAFs, such as the *Decision Making Impact Model*, the *Canadian Academy of Health Sciences (CAHS) model*, the *Hunter Medical Research Institute Framework to Assess the Impact of Translational health research* (*HMRI FAIT)* and the *Weiss Logic Model*, actively encourage specification of a line of sight to main anticipated benefits [60, 66, 95, 96]. This principle does not imply that a research project will realise the benefit, but explicitly encourages the researchers to articulate a pathway to impact (Fig. 1d). This requirement is applicable across the research spectrum e.g., T0-T4, for all needs-driven research and serves several purposes. First, it addresses the iMRIs’ concern that research should be directed by health needs and not solely by investigator-led curiosity [76]. Specification of the ‘need’, in consultation with consumers, can challenge commonly held perceptions of the outcome. For example, studies on patients suffering from Rheumatoid Arthritis found that fatigue, rather than pain dominated the patients’ research priorities [97]. This consideration would inform the focus of even basic scientists [76]. Appropriate indicators would be identified by the proponent research team to reflect engagement with and support from the ultimate intended beneficiary, the end (final) users, such as patient/consumer representatives, for their initiative.

The second value lies with the increased transparency to, and scrutiny of, the pathway to impact, and consequently, the identification of downstream issues that carry immediate implications for the proposed research project. For example, the need to address Diabetes mellitus type 2 is clearly established [98]. However, optimising the returns from HMR investment may not be realised through more research into the disease pattern physiology, but rather how to improve diets and increase exercise across the population [76]. Similarly, if adherence to clinical guidelines for a given health problem is low amongst primary care providers, modest improvements to the guidelines may not represent the optimal return on research investment [99]. Other examples demonstrate how the downstream issue might shape how the immediate research project is conducted. For example, if the pathway requires investment and progression of the initiative by the private sector (Fig. 1b), then the collection of requisite data for intellectual property is potentially relevant [76]. Similarly, synchronisation with existing government policy or programs may be critical for downstream translation [100].

Clarity regarding the potential pathway to impact will provide transparency to the steps, hurdles, and critical stakeholders along the anticipated pathway to impact. The research may ultimately follow a different path, but prospective identification of these issues will improve the potential relevance to end users, and the probability of translation for individual research projects. Identification of potential indicators at the inception of the research provides the mechanism to identify these issues and reporting of the realisation of these steps provides motivation for researchers to deliver.

### Inclusion of process metrics/indicators

The extensive time period between most HMR and eventual health impacts [101] was identified by the Institutes as a key issue for discovery science, but also clinical research and policy-relevant research [69, 76]. This represents a measurement issue both with respect to the time prior to which impacts could be acknowledged, and an attribution/causation issue, given potential confounding during this period. As a consequence, researchers cannot be accountable for, nor motivated by, the measurement of final impacts in the distant future.

The preceding principle outlines how the optimisation of the translation potential for a proposed project may account for multiple hurdles on the anticipated pathway to impact. Process metrics address this challenge by providing interim measures along the translation pathway. Accordingly, they provide for both the identification of key issues and the realisation of achievable goals along this pathway, for which a research project can be directly accountable. This principle is explicit within the *Process Marker Model*, the *TRO Performance Model*, *HMRI FAIT* and the *Balanced Scorecard* [60, 92, 101, 102].

Figure 1e demonstrates that the process metrics would be planned and captured for the stages from funding of the study, through conduct to the production of the research outputs. The indicators may relate to operational and research practice, e.g., the establishment of strategic plans, project resources, stakeholder engagement, ethics approval, power calculations for trial sample sizes, protocol papers, clinical trial registration, patient recruitment, results publications (irrespective of the effects), or the provision of data and code suitable for replication [103–105], or activities that address potential hurdles to translation, such as the attainment of specialist advice e.g. biostatistics/bioinformatics, health economics, financial/business case assessment, implementation science [76], etc., or other factors that may affect the probability of translation and impact [106, 107].

Ideally process metrics would be sourced from a list of standardised metrics to provide for subsequent inter-project and inter-institutional analysis. However, our prior qualitative research identified the concern that standard metrics could introduce perverse incentives that were not aligned with the optimisation of translation and impact [76]. The option to identify tailored metrics, in line with good practice [66], allows for potential conflicts, such as academic publication versus intellectual property protection, to be acknowledged and incentivised in line with the optimal translation pathway [39].

### A logic model component that embeds ‘next’ users and generates outcomes from outputs

This principle addresses the Institutes’ concern that process metrics may be insufficient to encourage actual translation [76]. One of the main inhibitors of research translation and impact from existing academic research relates to the acknowledgement of outputs, such as publications or grant success, as the endpoint for a research project. However, optimising productivity across all research will be driven by improvements in the probability that the results from each research project will be utilised by the next step on the pathway to impact. Research outputs are defined as the knowledge deliverables produced by research activity e.g. peer-reviewed papers, presentations, contributions to collaborative endeavours, education, etc. [60]. As demonstrated in Fig. 1f, research outputs transform to research outcomes/interim impacts following utilisation by a ‘next-user’ along the pathway to final impact [60, 83, 96]. This principle is derived from logic models, commonly used for program evaluation and represents a central tenet in the *Weiss Logic Model*, the *CAHS model*, the *Research Utilization Ladder*, *Decision Making Impact Mode, MCRI’s Research Translation and Impact Framework*, *HMRI FAIT* and Morton’s *Research Contribution Framework* [60, 83, 96, 108–110].

This principle necessitates that the definition of a ‘user’ is broadened to include both interim and final users of the research. Holistically, users are agents along the pathway to impact that utilise the research outputs, including the public sector, industry, and the community, but also other researchers [60]. Some funders prefer the term ‘next-user’ to reflect the interim step [111], leaving ‘final’ to reflect improvements in health, increases in economic output and employment, etc. This distinction maintains relevance from discovery through to implementation science.

The failure to engage users, both next and final users, at the inception stage of research was raised as an on-going problem for commercialisation, health system research, and policy research [76]. For example, the inability of a private pharmaceutical company to understand the contribution of a given piece of research, inhibits their ability to adequately assess the risk and consequently, inhibits their capacity to value intellectual property, even where value may legitimately exist [76]. Early engagement with users reduces the risks to relevance, comprehension, and implementation of the findings and applies equally to health systems and policy research, as commercialisation pathways.

As with process metrics, the value of this approach is that research outputs, users, and research outcomes accommodate research across the spectrum. For implementation science, clinical or health service research, patients may represent the users, in which case final impact may be assessed in health outcomes, quality of life measures, Quality-adjusted Life Years (QALYs) or similar. For clinical research, changes in clinical practice may represent an interim impact. For policy research, where timing of the political appetite for change is typically beyond researchers’ control, utilisation may reflect informing policy decisions makers, irrespective of final impact [76]. For discovery science, it is probable that other researchers represent a significant proportion of potential users. In this instance, peer-reviewed publications represent a measure of research output, and citation metrics within the peer-review literature may capture usage and impact upon other researchers. The utilisation of results data by other researchers e.g., for replication or meta-analyses, provide alternative measures of utilisation and interim impact. Industry represents another potential user of the research output from discovery science. In this instance, interim impacts would be measured by commercialisation arrangements, such as licences, technology-transfer agreements, etc.

In some circumstances the level of utilisation is unclear. For example, the exclusion of possible options [76] or the introduction of new paradigms [112] may impact upon clinical guidelines [76, 113] or policy outcomes [114], without necessarily being referenced in the documentation. This is not an all-encompassing solution. However, incorporation of this principle within an indicator-based approach incentivises researchers to prospectively engage with potential next-users, to identify appropriate indicators to reflect anticipated outputs and outcomes, and accordingly to improve the potential for translation. This principle consequently encourages co-production/co-creation for which there is good evidence that it improves the probability of translation of any given research project [104]. Indicators reflecting utilisation also reduce the incentive to generate unproductive outcomes e.g. worthless patents [115, 116]. while supporting the investigation of serendipitous opportunities, where an unanticipated pathway arises with greater potential for translation [117].

### Alignment of researchers’ incentives through integration within the research system

The outputs from prevailing research reflect the incentive structures within the existing research system [76, 118]. Most Australian iMRIs have an explicit mission to realise improvements in health [2]. However, the incentive frameworks for most the research, and researchers, facilitated by research institutes, are shaped by their employer e.g. universities or health services, and/or dictated by the requirements of the major external funding sources [76]. Furthermore, the revenue of research institutions relies significantly upon the grant funds and the research infrastructure funding tied to grant success. Consequently, while the institutions can incentivise translation-focussed research through the available levers, e.g., academic promotion criteria, equipment funding, etc., this influence may be minimal compared to the incentives inherent in the wider research system.

Incentives for translation and impact have gradually been introduced to the funding framework either at the institutional level e.g. UK REF [119], Australia’s EIA [120], or at the researcher level e.g. NHMRC’s Research Impact Track Record Assessment [121], but they are rarely purposely positioned with consideration to the existing incentive structures for researchers, and/or consideration of the mechanisms through which institutional incentives might transition to project-level research activity [57, 76, 118, 122]. For example, Australia’s EIA has only oblique influence on the direction and form of research at the coalface.

The incentives within the existing research system can also act to discourage translation. Examples exist of researchers that have concentrated upon research translation via clinical trials or commercialisation at the expense of traditional academic outputs [76]. However, as successful research passes into the private sector, minimal academic credit is provided for this initiative, the opportunity to win academic grants is accordingly reduced, research roles are not financially sustainable, and the translational experience of these researchers is lost to academia [76]. The challenge of duplication/replication represents a different example. The provision of data, methods, code and outcomes for independent scrutiny, beyond the requirements of peer review publication, presently represents a risk to career development, in case an error is found, rather than acknowledgement of transparency that improves the foundation for all dependent research [123]. If indicators credited independent duplication/replication, or the provision of information to enable duplication/replication, then this would increase the productivity from investment into HMR [124].

In summary, an indicator-based RIAF designed to engage researchers and incentivise optimisation of the return on investment must align with the exogenous financial and structural incentives within the existing research system. The prospective orientation of research represents one of the primary mechanisms to increase the productivity from individual research projects through identification of key indicators at the inception of the research. Figure 1c demonstrates that, from a researcher perspective, the grant application and review stage provides an existing incentive mechanism in which to incorporate these principles.

A number of funding schemes require extensive detail regarding the translation plans for proposed research, including the US CTSA [93], the UK NIHR Clinical Trials funding schemes [125], the NSW Cardiovascular Research Grants [126], NHMRC Partnership Program [127], and New Zealand’s Programme Research Grants [128]. Such translation plans could be readily extended to identify key indicators. However, most funding schemes do not require the specification of program logic models, or systems-based equivalents, including proposed indicators within research project plans.

Integration within the grant application process would achieve several goals. First, it would challenge research proposals to address maximisation of the translation and impact potential of their proposed research with equivalent rigour to the scientific rationale [76, 129]. Second, it would also allow for the selection process to identify proposals with a higher probability of translation. Third, it would replace the administrative burden to conduct RIA with the productive investment of researcher time to address the translational aspects of their proposals. Fourth, the collation of indicators within funding application portals would provide an efficient basis to report the outcomes, interim or final impacts from completed research projects, which is increasingly required by funders [111, 125], thereby building the evidence base regarding translation barriers, successes, etc. Finally, the dataset of reported indicators could be readily aggregated to enable queries/aggregation by institution, program or funding scheme.

Within Australia, the development of mechanisms to disseminate MRFF funding represents a critical opportunity to heighten the incentive for researchers and their administering institutions to prioritise research translation. The MRFF’s new funding schemes introduce elements of translation within the application process e.g., demonstration of consumer engagement [130], but the approach presently appears piecemeal rather than holistic, and there is no transparent framework to aggregate the data for subsequent *Analysis* or *Accountability*.

### Align with existing peer-review processes and norms

The peer-review process, while imperfect, remains the dominant and accepted method of research quality management [131]. Consequently, it is valuable for a RIAF to align, wherever appropriate, with these norms. RIAFs that do not incorporate this convention, such as the ARC’s *EIA*, the *Lean/Six-sigma* models, *Economic Impact Assessment* models, etc., may include an audit process, if sufficiently funded, but in the absence of peer review, they may not carry the confidence of the academic research community. Incorporation within existing research processes largely only occurs within existing RIAFs where the assessment process informs funding, most commonly at the institutional level. For example, the UK REF, while predominantly not founded on an indicator-based method, utilises peer-review to assess institutional performance [119].

Peer review is also important to select projects, with a higher probability of translation from the submitted proposals. The capacity for RIA to effectively maximise translation will be constrained without informed peer review. Grant review panels can be adjusted to include personnel with adequate knowledge to assess the quality of translation and impact proposals or capabilities (Fig. 1c). For example, many grant review panels have not possessed the capability to understand complex commercialisation issues. This peer-review process also provides a mechanism to drive best practice through the exposure of researchers to high- and low-quality proposals with respect to their research translation qualities.

Another potentially significant role also exists for peer-review at the reporting stage for funded projects. An existing mechanism for such review arises where the impacts from previous funded projects are reviewed and accounted for in subsequent funding applications e.g. Health Research Council of New Zealand’s Programme Grants [128]. In time, the development of natural language processing and machine learning may provide other more automated methods that could complement peer-review and make such reviews more comprehensive, objective and/or efficient [132].

### System (portfolio) thinking

The outlined principles seek to improve the probability that any given piece of funded research will be utilised by an anticipated ‘next’ user and potentially, with time, contribute to final health, societal or economic impacts. The contribution of any individual research project will vary to a greater or lesser extent, but incremental improvements to the probability of translation for each individual research project will lead to higher final impacts across the whole funding portfolio [133].

Over time, the database of indicators would provide for higher-quality analysis regarding the factors that contribute to translation e.g., co-production, factors that inhibit translation e.g., lack of commercial or consumer engagement, or factors that are ineffective, providing evidence for continual improvement of the application guidelines, etc. Similarly, the indicator database would provide data for the retrospective RIA of whole programs, funding portfolios, or institutions to meet the objective to provide *Accountability* for past funding and *Advocacy* for on-going commitments [92, 93]. The quality control process arising from integration with a peer-review process, would also serve to improve confidence in this information and reduce the cost of auditing and review.

## Discussion

A definitive objective represents a central premise in evaluation but is rarely specified within RIAFs [57]. This deficiency has potentially contributed to an on-going disconnection between the goals of research impact policy and academic debate regarding appropriate methods [56, 71]. The goals of research impact policies, and indeed the missions of most iMRIs [2], seek to optimise the productive impacts realised from the available investment into HMR. To this end, productivity should represent, one of, if not the primary, objective for RIAFs.

Indicator-based RIAFs reflect one of the most widely implemented approaches to RIA, but have been increasingly criticised, based on technical (e.g. cross-discipline consistency), theoretical (e.g. positivism, linear determinism) and data (e.g. bias towards measurable factors) limitations. These criticisms have validity but fail to prioritise the productivity objective in assessment of their relative merits.

The aim of this commentary was to demonstrate how the act of conducting an indicator-based RIA, not just its outcomes, can serve to optimise the impact from HMR. The process of identifying relevant indicators challenges assumptions and facilitates the refinement of research projects towards an initiative with an incrementally higher probability of producing interim impacts. For example, a prior systematic review and meta-analysis can demonstrate the requirement for further clinical trial evidence, and therefore, improve the probability that a further trial will generate impact [25]. Our prior studies found that RIA is predominantly about the incentives established by the RIAF, and the extent to which they align with the incentives within the prevailing research system [74–76, 134]. To optimise impact, the principles leverage and align with the incentives in the existing grant application and peer review processes.

The principles account for the main criticisms of indicator-based approaches. The challenge of retrospective attribution to specific research funding schemes, programs, or institutions is significant [38, 47, 68–71]. Prospective application and a focus on research projects within the control of researchers, provides for direct attribution and, where appropriate, causal association to the interim impacts generated from an individual study. The principles do not nullify criticism that the assessment of final impacts is subjective [55]. However, the requirement to identify an explicit ‘need’, preferably with consumers, and to engage ‘next’ users, ensures that the intended impacts, if achieved, are valued by the potential users/beneficiaries.

Academic freedom is partially constrained through a more prescriptive requirement for research to address societal needs, particularly if implemented through project funding application systems. However, there is nothing in the principles that dictates the research question, the approach to the research, placement across the research spectrum, the evolving direction of the research (i.e. serendipitous opportunities are encouraged), or the identification of the optimal translation pathway. As such, the principles retain most academic freedoms and accommodates the unpredictable nature of research [72, 73].

The problem of limited impact data has channelled much RIA toward bibliometrics or qualitative analysis [56]. The prospective identification of appropriate indicators potentially resolves this issue, provided an appropriate platform such as a grant application portal is available to efficiently collate the indicators. For researchers, utilisation of the application process replaces a stand-alone burdensome administrative obligation to assess their impact with a process that supports research teams to maximise the value of their research while collating relevant indicators.

The prioritisation of productivity in this commentary, does not imply that other objectives for RIA, such as *Advocacy* or *Accountability*, are not valid, nor that methods other than an indicator-based approach e.g. economic methods, qualitative research, realist approaches, cannot contribute to productivity. However, it is valuable to understand the merits of alternative methods for alternative objectives.

While prioritising productivity, the outlined approach can also provide data for *Accountability*, or *Advocacy*. Assuming integration within a grant application process that requires specification of indicators in line with the principles (within a program logic, systems-based, or realist evaluation model), and a reporting scheme that reconciles the results of the completed research, this data could be aggregated for these alternative objectives. Such a platform could also be utilised to prompt the collection of data for analytical insight. For example, the relevance of financial contributions from ‘next’ users, required in some current funding schemes, could be collected and assessed to examine whether this supports co-creation, utilisation of the research results, and the interim impacts generated. In this light, an indicator-based approach to RIA could contribute to a ‘learning’ research system capable of improving the evidence base regarding factors that support or undermine translation, distinguished for different stages of the research spectrum, and evaluating policies to continually optimise impact, that is, research on HMR.

This approach is consistent with the evaluators approach to the US CTSA program [93, 135], where an equivalent focus on the inception of research projects enables the impact assessment of research projects to focus upon prospective orientation, process monitoring and improvement, and shorter term outcomes/interim impacts, while impact assessment at the research institution, program, or funder level focus on longer term issues, analysis, management and retrospective insight [93, 135]. It is also consistent with the UK NIHR’s holistic approach to RIA where ‘impact fits into all stages of the research funding lifecycle from early-stage planning of research’ [136].

The approach to RIA outlined in this study is not without its limitations. First, the proposed principles are founded on the existing evidence, albeit limited, from our prior studies and within the wider academic and policy literature. The conjecture that this approach will improve the productive impacts/returns from investment in HMR is supported by anecdotal experience, but needs to be supported with observational or (quasi- or natural) experimental studies. Second, this approach only applies to consumer needs-driven research. It does not address the concerns of basic research scientists that RIA policy disadvantages curiosity-led research [60, 76]. If funding sources do not discriminate between needs-driven research and investigator-led discovery research, the incentives provided by an impact agenda, particularly through funding, may undervalue blue-sky curiosity-led research, potentially undermine productivity gains, and, at worst, risks incentivising ambit claims of attribution and scientific misconduct. Further research is required to examine whether RIA would be optimised by distinguishing different approaches for separate funding streams. Third, the complexity of translation pathways and the time-lag to final impacts is addressed through the focus on individual projects. However, this does not explicitly address the long-term impacts generated across a research career, nor Penfield, Baker [70]’s assertion of the subtler impacts, the knowledge creep, from research. Alternative approaches will be required to assess the contribution of researchers in this form.

## Conclusion

Optimisation of the productive impacts from investment into HMR represents the primary purpose of Research Impact policy. However, very few RIAFs explicitly prioritise productivity from which to determine the form, methods and content of the framework [94, 137]. This commentary demonstrates how the process of conducting an indicator-based RIA carries the potential to make an explicit contribution to the improvement of research translation and impact, and accordingly to the goal of research impact policy. The outlined principles seek to utilise the incentives generated by the process, to encourage researchers to address matters for which there is supporting evidence of their potential to improve the probability of translation, and thereby to generate improved impacts across a portfolio of funding into HMR. It is evident that this process could be effectively integrated within the research project grant funding procedure, particularly the application stage.

## Data Availability

Not applicable.

## Abbreviations

ARC EIA: Australian Research Council’s *Engagement and Impact Assessment*
CAHS: Canadian Academy of Health Sciences
CLAHRC: Collaborations for Leadership in Applied Health Research and Care (United Kingdom)
CTSA: Clinical and Translational Science Awards (United States)
HMR: Health and medical research
HMRI FAIT: Hunter Medical Research Institute Framework to Assess the Impact of Translational Health Research
iMRI: Independent Medical Research Institute
MCRI: Murdoch Childrens Research Institute
MRFF: Medical Research Future Fund (Australia)
NHMRC: National Health and Medical Research Council (Australia)
NSW: New South Wales (Australia)
NIHR: National Institute of Health Research (United Kingdom)
NSRC: National Survey of Research Commercialisation (Australia)
REF: Research Excellence Framework (United Kingdom)
RI: Research impact
RIA: Research Impact Assessment
RIAF: Research Impact Assessment Framework
